# Supplementary material for: Isotopic and microbotanical insights into Iron Age agricultural reliance in the Central African rainforest
Source: Commun Biol. 2020 Oct 27;3:619. doi: 10.1038/s42003-020-01324-2 (PMC7591565; doi:10.1038/s42003-020-01324-2)
Supplement: Supplementary file 1 — Supplementary Information [file 42003_2020_1324_MOESM1_ESM.docx]

**Supplementary Information for:**

**Isotopic and Microbotanical Insights into Iron Age agricultural reliance in the Central African rainforest**

Madeleine Bleasdale^1,2*^, Hans-Peter Wotzka^3^, Barbara Eichhorn^4+^, Julio Mercader^1,5^, Amy Styring^4,6^, Jana Zech^1^, María Soto^5^ , Jamie Inwood^5^, Siobhán Clarke^5^, Sara Marzo^1^, Bianca Fiedler^1^, Veerle Linseele^7^, Nicole Boivin^1,5, 8,9,^, Patrick Roberts^1,8*^

1. Department of Archaeology, Max Planck Institute for the Science of Human History, Kahlaische Straße 10, 07745 Jena, Germany
2. Department of Archaeology, University of York, King’s Manor, Exhibition Square, York, YO1 7EP
3. Institute of Prehistory, University of Cologne, Weyertal 125, 50931 Cologne, Germany
4. Institute of Archaeological Sciences, Goethe University, Norbert-Wollheim-Platz 1, D-60629 Frankfurt am Main, Germany
5. Department of Archaeology and Anthropology, University of Calgary, 2500 University Drive, N.W. Calgary, Alberta, T2N 1N4, Canada
6. School of Archaeology, University of Oxford, 1 South Parks Road, Oxford, OX1 3TG, United Kingdom
7. Department of Earth and Environmental Sciences, Center for Archaeological Sciences, University of Leuven, Celestijnenlaan 200E, 3001 Leuven, Belgium
8. Department of Archaeology, University of Queensland, St Lucia QLD 4072, Brisbane, Australia
9. Department of Anthropology, National Museum of Natural History, Smithsonian Institution, 10^th^ Street & Constitution Avenue, Washington, DC 20560, United States of America

+Deceased

*Correspondence to: Madeleine Bleasdale and Patrick Roberts
email: [bleasdale@shh.mpg.de](mailto:bleasdale@shh.mpg.de), [roberts@shh.mpg.de](mailto:roberts@shh.mpg.de)

This PDF file includes:

**Supplementary Note 1: Food production during the Central African Iron Age**

**Supplementary Note 2: Sites of study, chronology and new AMS dates**

**Supplementary Note 3: Reconstructing subsistence practices in tropical environments using stable isotopes**

**Supplementary Figure 1: Calibration of radiocarbon measurement for sample LON81/2 (SUERC-89286/GU53256)**

**Supplementary Figure 2: Human bone samples from Longa (LON) and Bolondo (BLD) showing the degree of organic preservation**

**Supplementary Figure 3: Supplementary Figure 3: Calibration curve for sample BLD 83/1 Individual 1 (SUERC-89287/GU53257)**

**Supplementary Figure 4: Calibration curve for sample BLD 83/1 Individual 2 (SUERC-89288/GU53258)**

**Supplementary Figure 5: Calibration curve for sample BLD 83/4 (SUERC-89289/GU53259)**

**Supplementary Figure 6: Calibration curve for sample BLD 83/5 (SUERC-89290/GU53260)**

**Supplementary Figure 7: Calibration curve for sample BLD 83/8 (SUERC-89291/GU53261)**

**Supplementary Figure 8: Tooth enamel results (M1-M3) for individual from MTNW**

**Supplementary Figure 9: Reference collection**

**Supplementary Table 1: Summary of previously published dates from IMB, LON and BLD**

**Supplementary Table 2: New radiocarbon dates for human burials from Longa (LON) and Bolondo (BLD)**

**Supplementary Table 3: δ^13^C and δ^15^N values for human and faunal bone collagen from BLD and LON.**

**Supplementary Table 4: δ^13^C and δ^18^O values for human and faunal tooth enamel from BLD, IMB, LON and MTNW**

**Supplementary Table 5: Summary table of number of starch granules and phytoliths observed from dental calculus samples from M1-M3 of the individual from MTNW.**

**Supplementary Table 6: δ^13^C and δ^15^N values of charred food remains from BLD**

**Supplementary Note 1: Food Production during the Central African Iron Age**

For decades, the palaeoenvironmental context for the spread of farming and Bantu-speaking groups across sub-Saharan Africa has been an important topic of debate ^1–4^. The spread of agriculture across Africa has been dominated by theories surrounding the “Bantu Expansion” and the palaeoenviromental conditions that coincided, and possibly facilitated, the migrations of these Bantu-speaking populations. Two significant palaeoenvironmental events have been argued to have fundamentally altered the vegetation and local ecosystems of the Central African rainforest during the Holocene. The first, around 4,000 cal. BP saw the contraction of rainforest along its northern and southern periphery, including the coastal regions of southern Cameroon and Gabon ^5,6^. The second phase, at *c.* 2,500 cal. BP, is characterised by the fragmentation and deforestation of the core evergreen rainforest zones (the Late Holocene rainforest crisis or LHRC), resulting in the rapid expansion of pioneer vegetation and creation of more open environments ^7^. Although these changes have been long discussed, they remain relatively poorly characterised and understood. Indeed, more recent palaeoenvironmental studies of lake basin records and marine cores have argued that, as this latter deforestation event was contemporaneous with the arrival of Bantu speaking groups, it could represent significant human alteration of the landscape, rather than natural forest retreat ^8–10^, with major implications for how we view the impact of early farmers in Central Africa. There have been, however, significant counter-arguments against a strong human impact on the forests during this phase, in particular, a discrepancy between timing of clear settlements and large-scale vegetation changes ^11,12^.

It has been proposed that the opening up of a ‘Savannah Corridor’ *c.* 2,500 cal. BP facilitated the movement of Bantu speaking farmers, from their hypothesised homeland at the present-day Nigeria-Cameroon border area, eastwards and southwards across the rest of the African continent ^3,10,13^. Since the opening up of the landscape has commonly been considered to have supported the introduction and cultivation of new crops, the equatorial forest of Central Africa has been viewed as a barrier for agricultural expansion. While agricultural practices became increasingly diverse and intensified in western Africa during the Iron Age, in the core forested regions of Central Africa small-scale food production, hunting, gathering, and fishing have been argued to have persisted ^14,15^. Linguistic models suggest the migration of Bantu speaking groups into the equatorial rainforest was delayed by as much as 300 years in comparison to rapid dispersals into savannah-type environments ^3^. Therefore the discovery of domesticated pearl millet (*Pennisetum glaucum),* a crop of Sahelian origin ^16,17^, at Early Iron Age sites in Cameroon ^18^ stimulated notions that its presence was indicative of more open environments and seasonal rainfall ^1,2^. There have, however, since been calls to re-examine the hypothesised environmental context for the early cultivation of pearl millet at sites in Central Africa ^20^.

Modern experiments have shown it can be successfully grown in the Inner Congo basin even during periods of high annual rainfall ^20^, challenging previously proposed environmental constraints for millet cultivation that form the basis of many broad models of farming expansion into Central Africa. The presence of pearl millet at Early Iron Age sites in Central Africa not only raises questions about the nature of the environmental setting in which it was cultivated but also about how communities were utilising this new crop. The paucity of domestic C_4_ cereal finds at African rainforest sites makes it challenging to explore this archaeologically however, and the importance of forest C_3_ crops, including yams and fruit trees such as *Canarium*, and of oil palm (*Elaeis guineeensis*) exploitation, should not be overlooked ^1,21–23^. Charred endocarps of *Canarium* *schweinfurthii* recovered from rockshelters in the Ituri rainforest attest to consumption of wild fruits throughout the Holocene ^24^. Oil palm endocarp remains in particular, are abundant at rainforest sites ^25^, and likely provided a major source of fat. Tubers such as wild and domesticated yams are still difficult to trace in archaeobotanical records though ethnographic evidence shows the importance and efficiency of wild yams exploitation for human nutrition in the Central African rainforest (e.g. ^26,27^), including species which also occur in the Inner Congo Basin. The potential role of domesticated yams during the Iron Age so far remains unresolved. Furthermore, archaeobotanical remains only show detailed snapshots of specific plants, in contrast, the isotope analysis of human and faunal tissue can provide an assessment of overall dietary reliance.

**Supplementary Note 2: Sites of study, chronology and new AMS dates**

All human bone samples dated in this study were sent to the Scottish Universities Environmental Research Centre AMS Laboratory, Glasgow (SUERC, Lab ID: GU). Radiocarbon ages were calibrated to calendar timescale using OxCal 4 ^28^ and IntCal13 atmospheric calibration curve ^29^.

**Imbonga**

Imbonga (IMB) is a waterside village located on the Momboyo River. It is the type-site for the earliest pottery tradition of the central equatorial rainforest. Several excavations were carried out here in the 1980s by Manfred Eggert ^30–32^, and a number of radiocarbon dates were published (Supplementary Table 1). For the present study, enamel was sampled from a human second molar discovered in sediment contained in a ceramic vessel at IMB 81/11 (Supplementary Table 1). It is a second molar of an individual between 9–12 years of age. No other skeletal remains were found in this context. The vessel, a richly decorated flat-based bowl attributable to the Early Iron Age Inganda style, had been found isolated but in spatial proximity to a number of Early Iron Age pottery deposits. Although possibly not *in situ* at discovery, the vessel was found upside down, i.e. in a position typical of Early Iron Age ritual pottery deposits known from the Inner Congo Basin. Two holes intentionally knocked into opposite sides of the vessel wall likewise suggest ceremonial burial. IMB 81/11 has not been directly dated. However, Inganda pottery is known to have been produced in the 2^nd^ and 1^st^ centuries cal. BC.

Assuming contextual integrity of the find, the tooth sample may therefore be regarded as by far the oldest evidence included in this study representing one of the earliest periods of the regional Early Iron Age, 200 years more recent, at the most, than regional Iron Age beginnings associated with Imbonga ceramics. Imbonga pottery has been found at some 60 sites across the western parts of the Inner Congo Basin. It is not associated with any stone artefacts ^32^ but with a sedentary way of life, an advanced iron metallurgy, and a food production system. Reconstructing the subsistence practices of these early pottery-making communities has been extremely challenging due to poor conditions of preservation and limited datasets. Charred pearl millet remains have been found in Imbonga period and slightly more recent Early Iron Age contexts at the sites of Iyonda on the Congo River and Boso-Njafo on the Lulonga River, dating between *c*. 2330–1960 BP ^33^. Other archaeobotanically documented contemporaneous food plants include cowpea and, possibly, tubers. It has also been hypothesised that yams and plantain were likely important staples during this time. However, the respective contributions of these plants to Early Iron Age human diets remain largely unknown.

**Longa**

Longa (LON) is situated on the Ruki River. Archaeological survey and excavations were carried out by Manfred Eggert in the 1970s and 1980s ^30,34^, and three radiocarbon dates were published (Supplementary Table 1). In the context of the present study, a bone fragment from one human individual (LON 81/2) was directly dated to between 1642 and >1938 cal AD (95% probability) (Supplementary Figure 2; Supplementary Table 2).

**Bolondo**

Bolondo (BLD) is situated on the floodplain of the Tshuapa River, within the zone of seasonal inundation. Today, it is a small, year-round fishing camp inhabited by a few families who live in lightweight houses erected on artificial clay and refuse mounds. According to ethnography (e.g. ^35^) such camps (locally called *nganda*) used to be seasonally inhabited special-function sites, each associated with a particular nearby dryland village where only a tiny fraction of the total population would be present at the pertaining *nganda* during the low-water fishing season. However, due to recent overpopulation and food shortage at urban centres, that has created new demands and sales chances for smoked fish, a great many *nganda* have shifted from seasonal subsistence fishing to perennial inhabitation and increasingly market-oriented fishing. In any case, freshwater fish has remained a conspicuously dominant foodstuff for *nganda* residents to the present day.

Excavations at the site first took place in 1983 as part of Manfred Eggert’s former River Reconnaissance Project, with further excavations, pollen coring, and new radiocarbon dating in 2016 financed by the *Deutsche Forschungsgemeinschaft* ^32^. Excavations have revealed a neatly stratified sequence from *c*. cal. years AD 1330 to today (Supplementary Table 1) featuring house remains, stake holes, midden deposits, and a number of human inhumation burials as well as botanical and faunal remains. Animal bones include mainly freshwater fish ^36^ but also crocodile, antelope, domestic dog and goat ^37^. Most significant among the archaeobotanical finds are charred remains of Pearl millet (*Pennisetum* *glaucum*) and banana phytoliths.

The human and faunal remains in this study came from excavations in 1983 (BLD 83/*) and 2016 (BLD 16/*). Due to the waterlogged conditions, there was good overall organic preservation (Supplementary Figure 2). For stable isotope analysis, bones and tooth enamel were sampled from 11 human individuals. While it was impossible to obtain both a bone and enamel sample from every individual there is direct crossover between the two sets of tissues for five individuals.

For five individuals buried at Bolondo it was possible to obtain new direct AMS dates from bone collagen (Supplementary Table 2). While calibration plateaus impede precise calendar dating in all instances but one (SUERC-89290, BLD 83/5), it appears this individual in addition to 83/1 individual 2 and BLD 83/4 are from an earlier period and burials 83/1 and 83/8 are from a later period (Supplementary Figures 3-7). Considered together, the entire suite of dates may be regarded as representing a time span between the second half of the 15^th^ and the early 19^th^ century cal AD. Therefore, representing pre-colonial funerary evidence well before imperial regulations widely inhibited human interment at fishing camps.

None of the Bolondo burials were associated with ceramic finds interpretable as grave goods. However, all of them were found in stratigraphic contexts representing intermediate and recent layers of the site stratigraphy. These are characterised by settlement refuse including large quantities of pottery fragments belonging to the following style groups of the regional Later Iron Age Tshuapa Tradition (in stratigraphic sequence from earliest to most recent): Bolondo, Bokone, Bolombi, and Ilemba-Bokonda ^32^.

**Matangai Turu Northwest**

Matangai Turu Northwest (MTNW) is a granite rock shelter with a floor area of 47 m^2^ located in the Ituri region, under a mixed, lowland rainforest. Mercader and colleagues ^38^ retrieved a human skeleton from level five, in a sandy clay loam with slightly acidic pH, 5% water content, and directly dating to 813 ± 35 ^14^C BP (1218-1277 AD, 1 sigma: UtC-5074). This same level yielded charcoal fragments and endocarps from Guineo-Congolian trees such as *Canarium schweinfurthii* (Burseraceae) and the African oil palm (*Elaeis guineensis*, Arecaceae) ^39^. From a faunal perspective, all taxa contemporaneous with the burial are forest taxa ranging from snails (Achatina, Limicolaria) to bovids, primates, rodents, and carnivores. Unfortunately, none of this fauna could be located, and thus sampled, for this study. No domesticated fauna was found, and the overall palaeoenvironment at the time of occupation, as established by phytolith analysis, is a closed-canopy forest ^40^.

Dental pathologies include dental calculus and enamel hypoplasias but no caries. Previously published results report intentional incisor mutilation ^38^, a practice known among local agriculturalists. The stature of the buried individual averaged 156-159 cm not allowing us to conclude if this person was Mbuti-related. Contextually, the human remains associate with LSA lithics ^41^, Late Iron Age ceramics stylistically connected to those from the Western branch of the East African Rift System ^42^, and one isolated iron bar, without evidence of smelting or forging ^43^.

**Supplementary Note 3: Reconstructing subsistence practices in tropical environments using stable isotopes**

The stable light isotope analysis of human and animal tissues and food residues are widely applied methodologies for palaeodietary reconstruction ^44–46^. δ^13^C and δ^15^N analysis of bulk bone collagen is one of the most commonly applied approaches, and enables exploration of the degree to which humans were reliant on plants following the two main photosynthetic pathways (C_3_ and C_4_), and their animal consumers, as well as their position within the foodchain. The archaeological sites of this study currently represent tropical forest environments largely dominated by C_3_ forest species, as well as oil palm (*Elaeis guineensis)*. Phytolith evidence for MTNW has confirmed a similar environmental context (closed canopy forest) was present at the time of occupation ^40^. These C_3_ plants that dominate tropical forest environments, as well as domesticated oil palm and yams, have a δ^13^C between -35‰ and -19‰ making them distinguishable from C_4_ plants, which include wild grasses, millets and sorghum, which have a δ^13^C between -13‰ to -8‰ ^47–50^. C_3_ plants growing under a closed canopy, as well as their consumers, have even lower δ^13^C due to low light and recycled the CO_2_ the well-documented so-called ‘canopy effect’ ^51,52^. The distinct δ^13^C of these groups of plants is passed into the tissues of their consumers with a known fractionation effect ^54^. δ^15^N of bone collagen provides information relating to the trophic level of consumers, being elevated *c.* 3-5‰ with each step in a foodchain ^54^. Longer food chain lengths in aquatic systems mean that aquatic resources tend to also have higher δ^15^N values, enabling their distinction from terrestrial resources – something that can be difficult on the basis of δ^13^C alone ^55,56^.

The δ^13^C of bone collagen largely reflects protein intake averaged over a number of years and consequently plant consumption can be under-represented ^53,57,58^. In contrast, δ^13^C of the hydroxyapatite of tooth enamel is more reflective of the whole diet of an individual (proteins, fats, and carbohydrates) and can provide dietary information at the time of tooth development that will vary depending on the tooth sampled ^59^. For example, while femur bone collagen δ^13^C represents approximately the last 10 years of protein intake in the diet ^58^, the tooth enamel δ^13^C of human 3rd molars is influenced by the whole diet intake between 9-16 years of age ^60^. Tooth enamel also offers the opportunity to measure δ^18^O that reflects water and food sources, and can provide insight into the broader environment experienced by an individual including evaporative potential and, indirectly, insights into canopy density ^61,62^. The primary influence on tooth enamel δ^18^O will depend on whether the animal studied is an obligate drinker (gets the majority of its water from open water sources) or a non-obligate drinker (gets a major portion of water from consumed foods) ^63^. Tooth enamel has been the material of choice for palaeodietary studies in the tropics due to its high levels of preservation, and the fact that dietary contributions of low protein plant resources are more visible ^64–66^.

The stable isotopic analysis of human tissues can provide insights into the overall reliance of an individual on different food groups, while the analysis of food or charred foodstuffs is more likely to provide information about subsistence practices at a community or site-level. Integrated isotopic approaches using compound-specific carbon isotopes and bulk stable isotopes have detected C_4_ crops, such as millet and maize, in archaeological ceramics ^67–69^. However, the samples in this study were isolated finds of charred food fragments recovered through flotation and have no direct association with ceramics. In recent years, there has been growing recognition that such charred foodstuffs have great potential for providing insights into diet, food processing and cooking, with foodstuffs such as bread, doughs or porridge being identified in the archaeological record ^70,71^. The δ^13^ results in this study suggest that two of the food fragments with visible millet remains indeed originate from pearl millet pointing to cereal-based food, whereas the others with a smoother vesicular porridge-type texture yielded C_3_ signals pointing to non-cereal food.

**
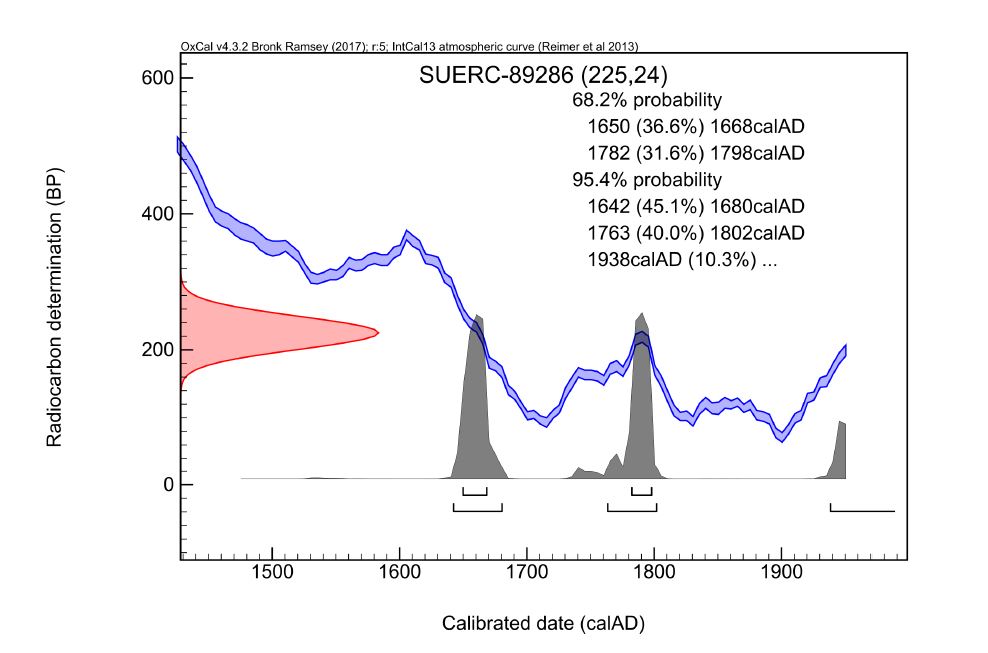
**

**Supplementary Figure 1: Calibration of radiocarbon measurement for sample LON81/2 (SUERC-89286/GU53256)**


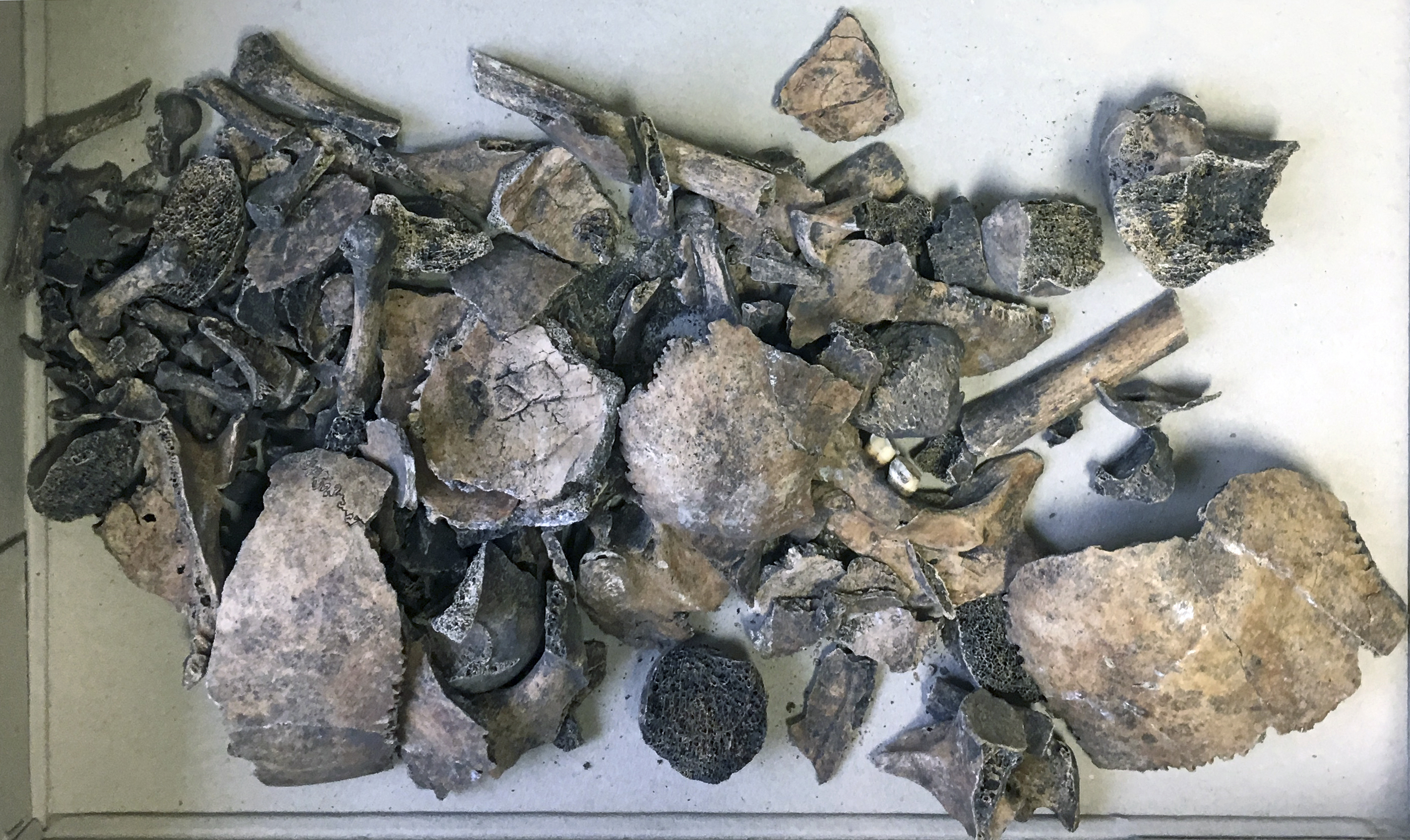

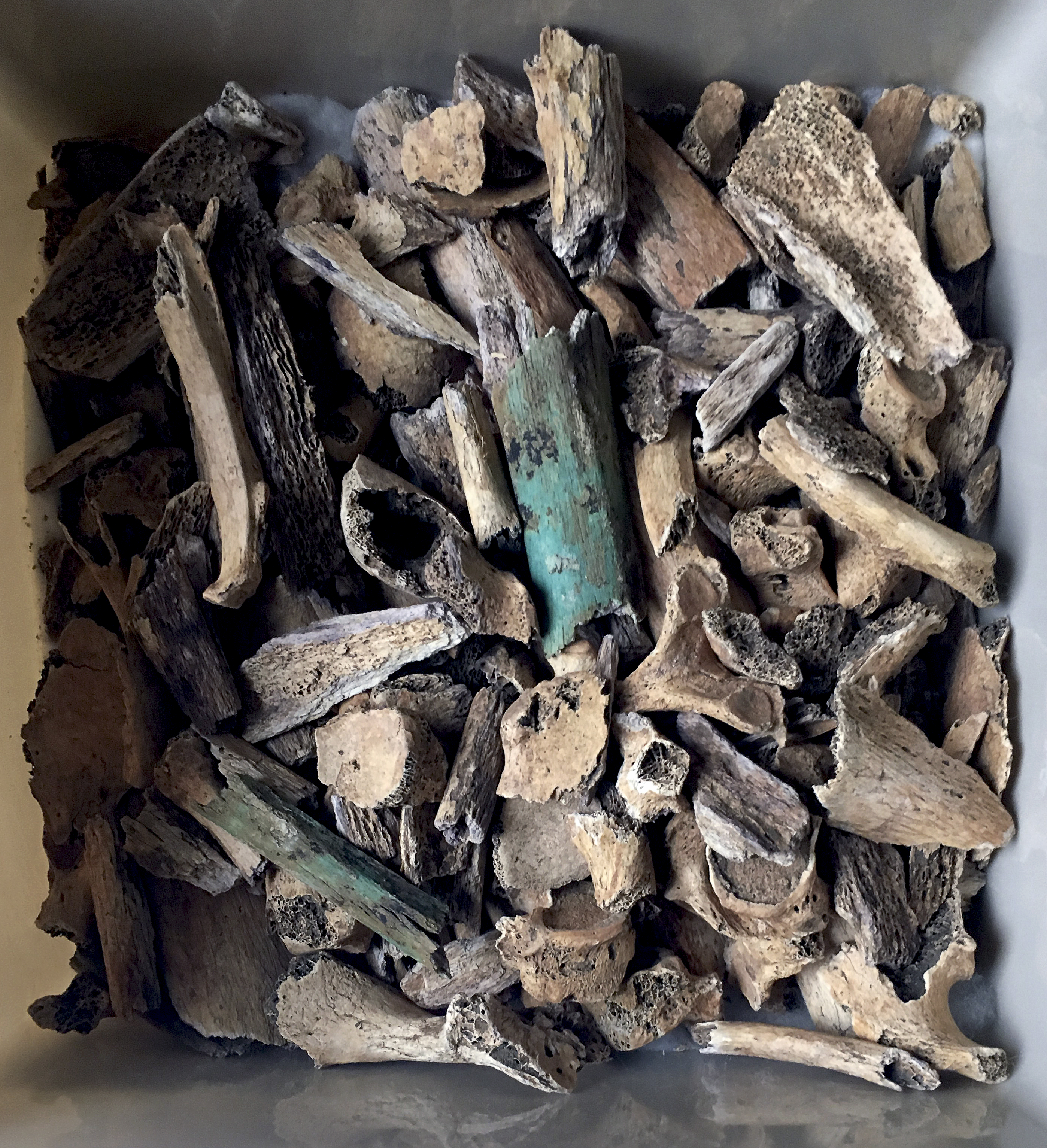


**Supplementary Figure 2:** **Human bone samples from Longa (LON) and Bolondo (BLD) showing the degree of organic preservation**

Left: Part of the postcranial skeletal remains from LON 81/2. Right: The bulk of the human bones from BLD 83/4.

**
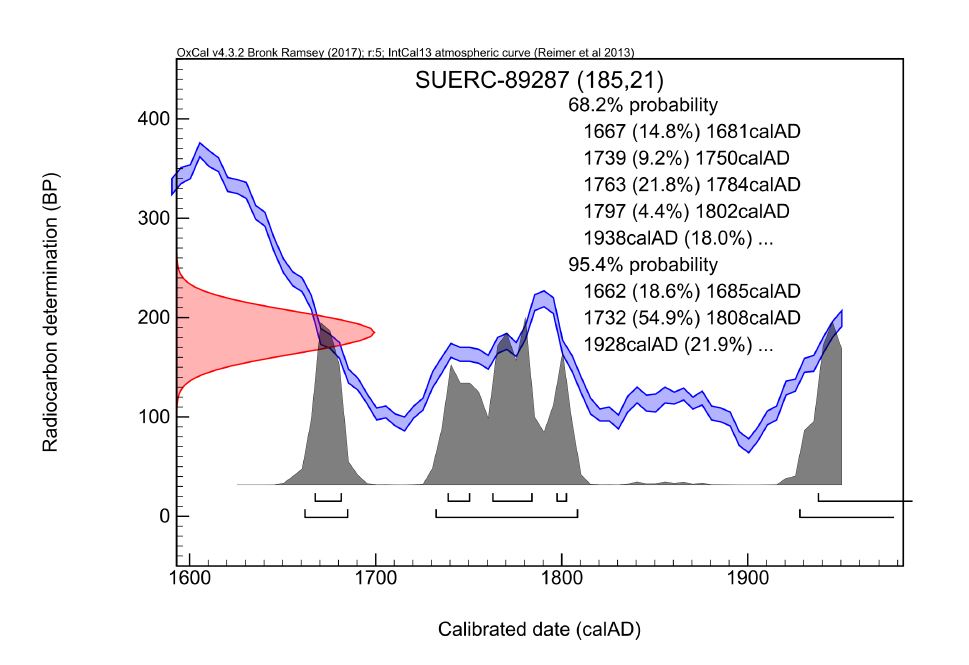
**

**Supplementary Figure 3: Calibration curve for sample BLD 83/1 Individual 1 (SUERC-89287/GU53257)**

**
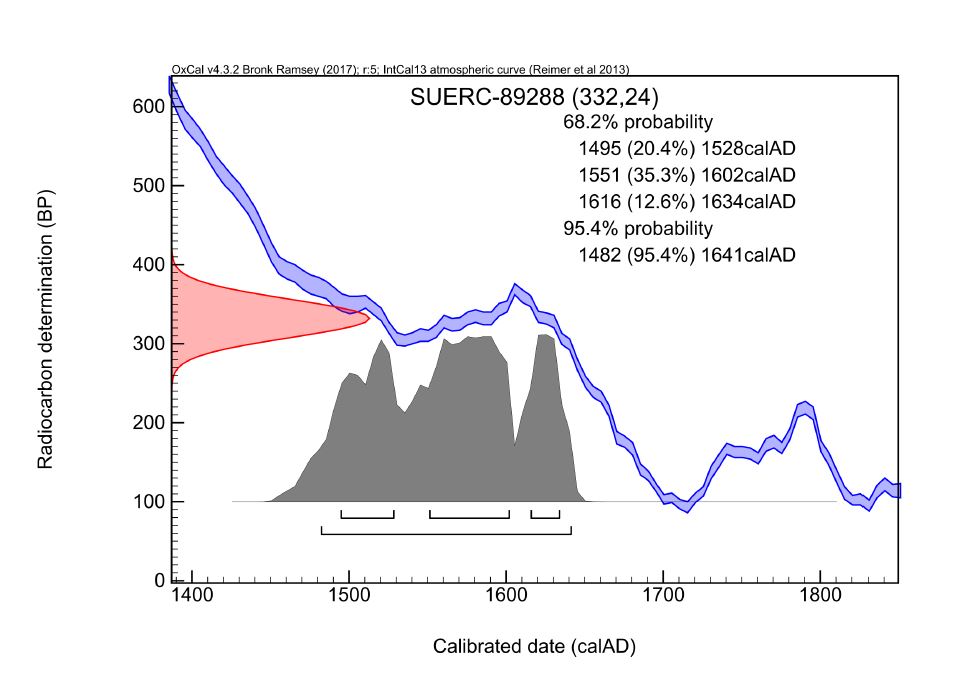
**

**Supplementary Figure 4: Calibration curve for sample BLD 83/1 Individual 2 (SUERC-89288/GU53258)**

**
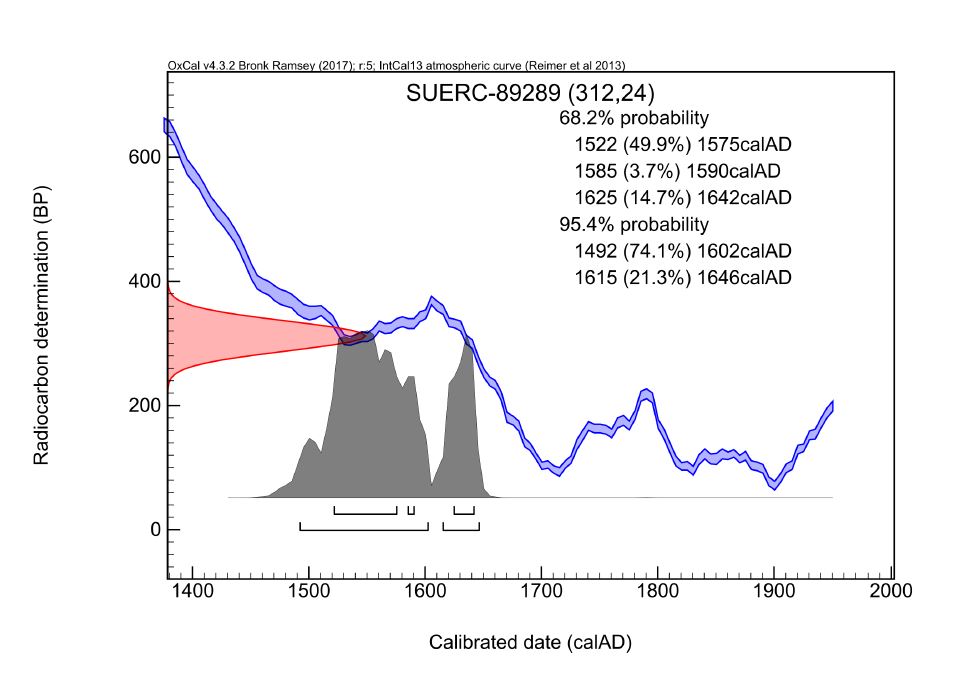
**

**Supplementary Figure 5: Calibration curve for sample BLD 83/4 (SUERC-89289/GU53259)**

**
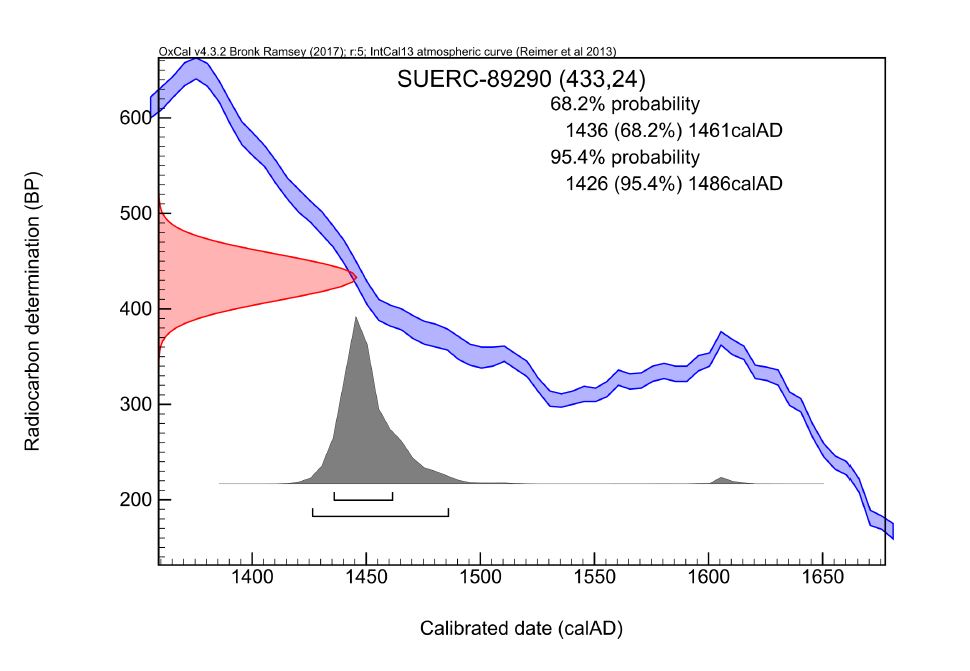
**

**Supplementary Figure 6: Calibration curve for sample BLD 83/5 (SUERC-89290/GU53260)**

**
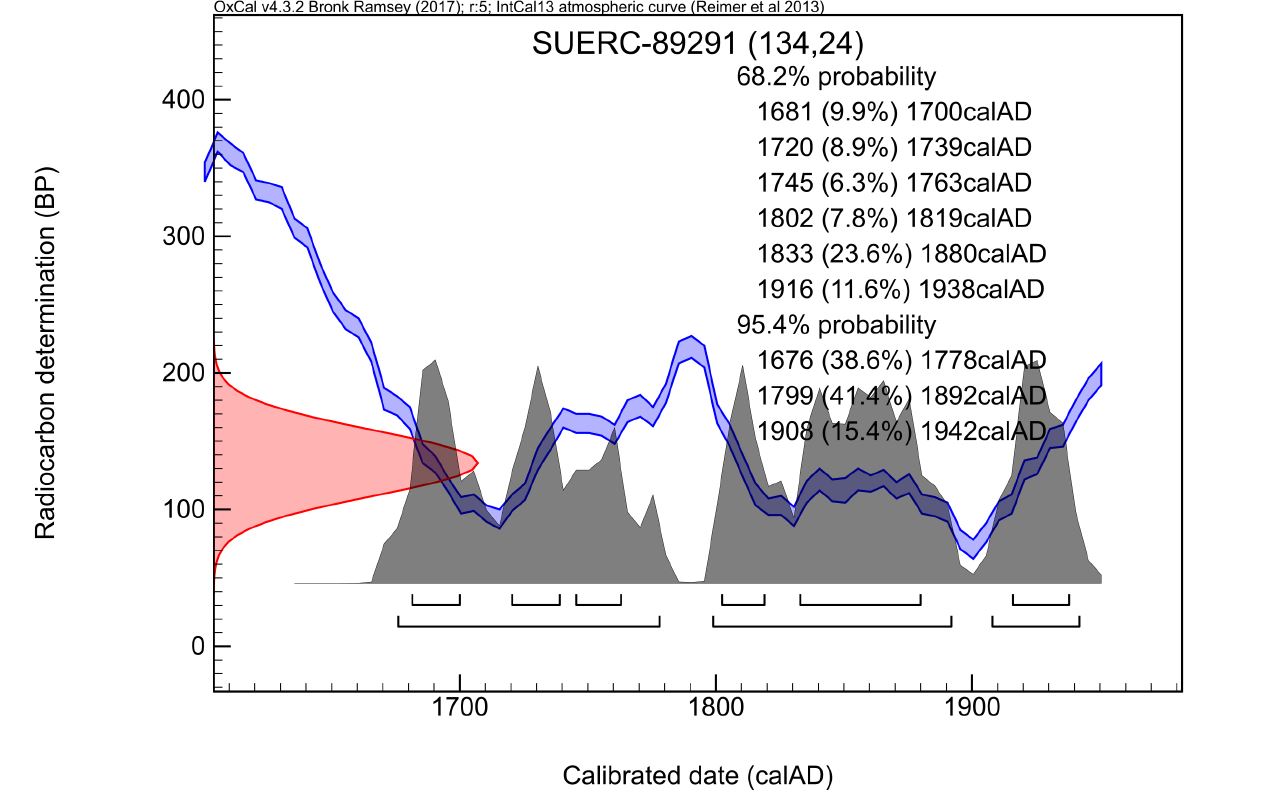
**

**Supplementary Figure 7: Calibration curve for sample BLD 83/8 (SUERC-89291/GU53261)**


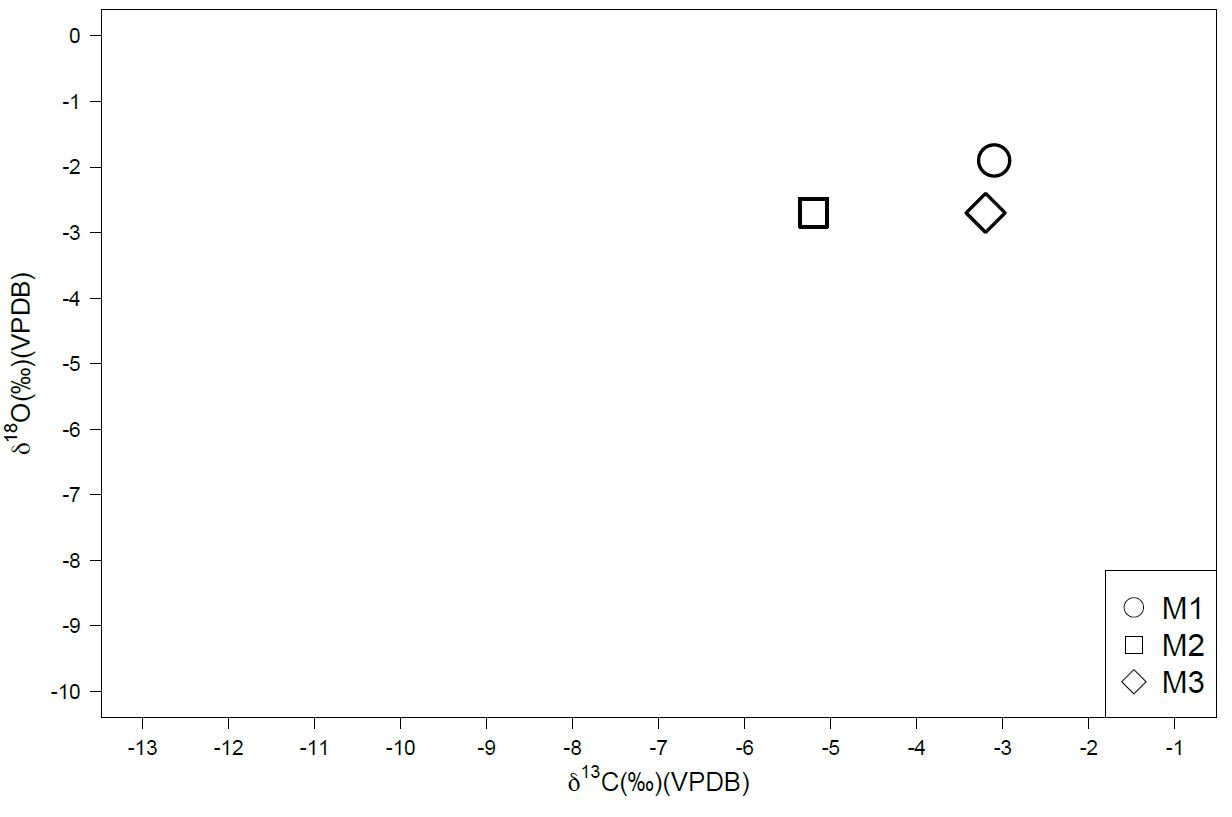


**Supplementary Figure 8: Tooth enamel results (M1-M3) for individual from MTNW**

**Supplementary Figure 9: Reference collection**

Cereal starch: a) *Eleusine coracana*, b) *Eleusine africana*, c) *Pennisetum glaucum*, d-h) *Sorghum bicolor.* Phytoliths*:* i-n) *Elaeis guineensis* (nutshell).

**Supplementary Table 1: Summary of previously published dates from IMB, LON and BLD.**

(GrN: Groningen, Laboratory for General Physics. Hv: Hannover, Lower Saxony State Office for Soil Research. KI: Kiel, C14-Laboratory of the Institute for Pure and Applied Nuclear Physics. KN: Universität Köln, Labor für C14-Datierung, Institut für Ur- und Frühgeschichte. – Note that Hannover (Hv) results are considered unreliable ^32,72^.

| **Site** | **Context** | **Material** | **Radiocarbon date** | **Calibrated date (95%)** | **Lab no.** |
| --- | --- | --- | --- | --- | --- |
| Imbonga | IMB 81/3 | Charcoal | 2900 ± 285 BP | 1450–800 cal BC | Hv-11576 |
|  | IMB 83/1 | Charcoal | 2665 ± 110 BP | 920–790 cal BC | Hv-12614 |
|  | IMB 81/9/1 | Charcoal | 2160 ± 90 BP | 375–100 cal BC | KI-2428 |
|  | IMB 81/9/1 | Charcoal | 3775 ± 105 BP | 2453–2039 cal BC | Hv-11574 |
|  | IMB 81/1 | Charcoal | 2860 ± 280 BP | 1420–800 cal BC | Hv-12207 |
|  | IMB 81/1 | Charcoal | 2130 ± 125 BP | 380–10 cal BC | Hv-11575 |
| Longa | LON 81/1 | Charcoal | 730 ± 75 BP | 1236–1289 cal AD | Hv-11571 |
|  | LON 81/1 | Charcoal | 500 ± 90 BP | 1322–1454 cal AD | GrN-13586 |
|  | LON 81/1 | Charcoal | 260 ± 120 BP | 1470–1955 cal AD | KN-4205 |
| Bolondo | BLD 83/1 | Charcoal | 1725 ± 95 BP | 213–416 cal AD | Hv-12624 |
|  | BLD 83/2 | Charcoal | 1195 ± 70 BP | 720–896 cal AD | Hv-12619 |
|  | BLD 83/2 | Charcoal | 1175 ± 210 BP | 650–1030 cal AD | Hv-12618 |
|  | BLD 83/1 | Charcoal | 915 ± 105 BP | 1010–1230 cal AD | Hv-12625 |
|  | BLD 83/1 | Charcoal | 660 ± 80 BP | 1270–1395 cal AD | GrN-13078 |
|  | BLD 83/2 | Charcoal | 230 ± 110 BP | 1514–1955 cal AD | KN-4203 |

**Supplementary Table 2: New radiocarbon dates for human burials from Longa (LON) and Bolondo (BLD)**

Dates provided by the Scottish Universities Environmental Research Centre (SUERC), University of Glasgow. Calibrated date ranges were obtained by means of the Oxford Radiocarbon Accelerator Unit calibration program OxCal v4.3.2 ^28^ using the IntCal13 atmospheric calibration curve ^29^

| **Context** | **Material** | **Radiocarbon date** | **Calibrated date (95%)** | **Lab no.** |
| --- | --- | --- | --- | --- |
| LON 81/2 | Bone | 225 ± 24 BP | 1642–after 1938 cal AD | SUERC-89286 (GU53256) |
| BLD 83/1, Indv. 1 | Bone | 185 ± 21 BP | 1662–after 1928 cal AD | SUERC-89287 (GU53257) |
| BLD 83/1, Indv. 2 | Bone | 332 ± 24 BP | 1482–1641 cal AD | SUERC-89288 (GU53258) |
| BLD 83/4 | Bone | 312 ± 24 BP | 1492–1646 cal AD | SUERC-89289 (GU53259) |
| BLD 83/5 | Bone | 433 ± 24 BP | 1426–1486 cal AD | SUERC-89290 (GU53260) |
| BLD 83/8 | Bone | 134 ± 24 BP | 1676–1942 cal AD | SUERC-89291 (GU53261) |

**Supplementary Table 3: δ^13^C and δ^15^N values for human and faunal bone collagen from BLD and LON.**

*indicates samples that were not carried forward for interpretation as %N was <5% and %C was <15% indicating poor sample quality.

| **Sample ID** | **Site** | **Calibrated Dates (95% confidence)** | **Species** | **Element** | **δ^13^C‰**  **VPDB** | **%C** | **δ^15^N‰ AIR** | **%N** | **C/ N** |
| --- | --- | --- | --- | --- | --- | --- | --- | --- | --- |
| BLD 16/5-46 HV | BLD |  | Bichir (*Polypterus* sp.) | Vertebra | -24.7 | 41.5 | 12.7 | 13.4 | 3.6 |
| BLD 16/5-53 | BLD |  | *Crocodylidae* | Tibia | -22.6 | 38.5 | 11.1 | 13.8 | 3.3 |
| BLD 16/5-55 | BLD |  | *Crocodylidae* | Femur | -23.3 | 36.6 | 11.4 | 13.0 | 3.3 |
| BLD 16/5-71 | BLD |  | Catfish (*Clarias* sp.) | Pectoral spine | -23.0 | 39.8 | 9.4 | 13.5 | 3.5 |
| BLD 16/5-53 | BLD |  | *Canis familiaris* | Mandible | -19.9 | 41.5 | 12.3 | 14.9 | 3.3 |
| BLD 16/5-13 | BLD |  | *Capra hircus* | Scapula | -19.7 | 38.8 | 9.9 | 13.9 | 3.3 |
| BLD 83/2-8 | BLD |  | *Sylvicapra grimmia* | Metacarpus | -23.7 | 36.5 | 5.9 | 12.4 | 3.4 |
| BLD 83/2-6+7 | BLD |  | Small antelope | Tibia | -22.5 | 28.9 | 7.3 | 9.8 | 3.4 |
| BLD 83/2-10 | BLD |  | Fox-sized carnivore | Humerus | -20.3 | 24.3 | 13.7 | 8.0 | 3.5 |
| BLD 83/2-10 | BLD |  | *Capra hircus* | Mandible | -18.2 | 43.6 | 9.8 | 15.8 | 3.2 |
| LON 81/2 | LON | 1642**–**1938 cal AD | *Homo sapiens* | Rib | -21.8 | 24.2 | 15.1 | 8.7 | 3.2 |
| BLD 16/5-35 | BLD |  | *Homo sapiens* | Rib | -16.7 | 33.7 | 13.6 | 12.0 | 3.3 |
| BLD 16/5-31 | BLD |  | *Homo sapiens* | Metatarsal | -16.3 | 39.6 | 13.4 | 14.2 | 3.2 |
| BLD 83/4 | BLD | 1492**–**1646 cal AD | *Homo sapiens* | Scapula | -17.5 | 28.5 | 13.6 | 10.4 | 3.2 |
| BLD 83/3: Individual 2 | BLD |  | *Homo sapiens* | Bone fragment | -21.0 | 42.4 | 16.9 | 15.6 | 3.2 |
| BLD 83/6: Individual 2 | BLD |  | *Homo sapiens* | Metacarpal | -18.8 | 29.5 | 14.3 | 10.5 | 3.2 |
| BLD 83/1: Individual 2 | BLD | 1482**–**1641 cal AD | *Homo sapiens* | Rib | -16.5 | 35.7 | 14.7 | 13.4 | 3.1 |
| BLD 83/1: Individual 1 | BLD | 1662**–**1928 cal AD | *Homo sapiens* | Rib | -17.4 | 40.5 | 15.7 | 15.1 | 3.1 |
| BLD 83/5 | BLD | 1426**–**1486 cal AD | *Homo sapiens* | Femur | -18.8 | 25.0 | 14.2 | 9.2 | 3.2 |
| BLD 83/8 | BLD | 1676**–**1942 cal AD | *Homo sapiens* | Long bone | -20.2 | 26.5 | 14.2 | 9.7 | 3.2 |
| BLD 83/10 | BLD |  | *Homo sapiens* | Rib | -16.7 | 29.0 | 13.5 | 10.4 | 3.2 |
| BLD 83/6/I | BLD |  | *Homo sapiens* | Bone fragment | -18.6 | 32.2 | 15.7 | 11.5 | 3.3 |
| *BLD 83/6 Individual 1 | BLD |  | *Homo sapiens* | Rib | -19.6 | 10.4 | 12.9 | 3.5 | 3.5 |
| *BLD 83/7 | BLD |  | *Homo sapiens* | Rib | -17.1 | 10.2 | 14.0 | 3.4 | 3.3 |

**Supplementary Table 4: δ^13^C and δ^18^O values for human and faunal tooth enamel from BLD, IMB, LON and MTNW**

| **Sample ID** | **Site** | **Species** | **Tooth** | **δ^13^C‰**  **VPDB** | **δ^18^O‰**  **VPDB** |
| --- | --- | --- | --- | --- | --- |
| IMB 81/11 | Imbonga | *Homo sapiens* | M2 | -14.1 | -3.5 |
| LON 81/2 | Longa | *Homo sapiens* | M3 | -15.1 | -2.8 |
| MTNW | Matangai Turu Northwest | *Homo sapiens* | M3 | -3.2 | -2.8 |
| BLD 83/7, Individual 2 | Bolondo | *Homo sapiens* | M3 | -12.0 | -2.6 |
| BLD 83/7, Individual 1 | Bolondo | *Homo sapiens* | M3 | -11.2 | -2.4 |
| BLD 83/4-2 | Bolondo | *Homo sapiens* | M2 | -14.7 | -2.8 |
| BLD 83/3, Individual 2 | Bolondo | *Homo sapiens* | M3 | -11.7 | -2.9 |
| BLD 83/3, Individual 1 | Bolondo | *Homo sapiens* | M3 | -11.6 | -2.6 |
| BLD 83/6, Individual 1 | Bolondo | *Homo sapiens* | M2 | -12.0 | -2.3 |
| BLD 83/6, Individual 6 | Bolondo | *Homo sapiens* | M2 | -11.2 | -4.9 |
| BLD 83/1, Individual 1 | Bolondo | *Homo sapiens* | M3 | -13.6 | -3.2 |
| BLD 83/8 | Bolondo | *Homo sapiens* | M2 | -11.4 | -3.6 |
| BLD 83/10 | Bolondo | *Homo sapiens* | M3 | -10.8 | -4.1 |
| BLD 16/1-13 | Bolondo | *Homo sapiens* | M3 | -11.5 | -3.5 |
| BLD 16/5-53, Bot ID 394 | Bolondo | *Canis familiaris* | Molar | -13.6 | -2.7 |
| BLD 83/2-10 | Bolondo | Fox-sized carnivore | Molar | -10.7 | -2.3 |
| BLD 83/2-10, No. 7 | Bolondo | *Capra hircus* | Molar | -9.6 | -1.0 |
| BLD 16/5-71, Bot ID 412 | Bolondo | Lungfish *(Protopterus* sp.*)* | Upper jaw | -13.7 | -4.3 |
| BLD 16/5-46, HV Bot ID 383 | Bolondo | Catfish *(Clarias* sp.*)* | Upper jaw | -11.1 | -7.4 |
| BLD 16/5-46, HV Bot ID 383 | Bolondo | Catfish *(Clarias* sp.*)* | Upper jaw | -11.5 | -6.3 |

**Supplementary Table 5: Summary table of number of starch granules and phytoliths observed from dental calculus samples from M1-M3 of the individual from MTNW.**

|  |  | **M1** | **M2** | **M3** | **Total** |
| --- | --- | --- | --- | --- | --- |
| **Starch** | Parabolic | 0 | 8 | 1 | 9 |
|  | Ovate | 0 | 1 | 0 | 1 |
|  | Oblong elongate | 1 | 0 | 1 | 2 |
|  | Polygonal | 3 | 4 | 1 | 8 |
|  | Orbicular | 6 | 7 | 2 | 15 |
|  | Quadratic | 0 | 3 | 0 | 3 |
| **Phytolith** | Globular tuberculate/echinate | 9 | 0 | 0 | 9 |

**Supplementary Table 6: δ^13^C and δ^15^N values of charred food remains from BLD**

*N_2_ peak was too small for reliable determination of δ^15^N values

| **Sample ID** | **Site** | **Archaeological ID** | **Botanical ID** | **δ^13^C‰**  **VPDB** | **%C** | **δ^15^N‰ AIR** | **%N** | **C/N** |
| --- | --- | --- | --- | --- | --- | --- | --- | --- |
| BE01 | Bolondo | BLD 16/1-41 | 377 | -27.9 | 51.2 | *4.7** | 0.9 | 69.9 |
| BE02 | Bolondo | BLD 16/1-38 | 371 | -27.7 | 68.4 | *2.6** | 0.6 | 138.6 |
| BE03 | Bolondo | BLD 16/1-39 | 373 | -28.7 | 51.6 | *6.3** | 0.6 | 108.1 |
| BE04 | Bolondo | BLD 16/1-36 | 367 | -28.7 | 51.6 | *4.2** | 0.6 | 94.2 |
| BE05 | Bolondo | BLD 16/1-37 | 369 | -28.4 | 38.4 | *3.9** | 0.6 | 75.4 |
| BE06 | Bolondo | BLD 16/5-61 | 402 | -9.3 | 42.0 | 7.8 | 2.5 | 19.7 |
| BE07 | Bolondo | BLD 16/5-59 | 400 | -9.3 | 42.0 | *7.1** | 2.5 | 20.0 |
| BE08 | Bolondo | BLD 16/5-59 | 400 (2) | -24.1 | 31.2 | *8.4** | 1.5 | 24.8 |

**Supplementary References**

1. [Höhn](http://paperpile.com/b/iTMxNF/eqy3), A., Kahlheber, S., Neumann, K. & Schweizer, A. Settling the rain forest: the environment of farming communities in southern Cameroon during the first millennium BC. in [*Dynamics of Forest Ecosystems in Central Africa during the Holocene: Past-Present-Future.* (ed. Runge, J.) 29–41 (Taylor and Francis, London, 2007).](http://paperpile.com/b/iTMxNF/eqy3)

2. Kahlheber, S., Bostoen, K. & Neumann, K. Early plant cultivation in the Central African rain forest: first millennium BC pearl millet from South Cameroon. [*Journal of African Archaeology* **7**, 253–272 (2009).](http://paperpile.com/b/iTMxNF/eFqO)

3. [Grollemund, R. *et al.*](http://paperpile.com/b/iTMxNF/0Noxz) Bantu expansion shows that habitat alters the route and pace of human dispersals. [*Proc. Natl. Acad. Sci. U. S. A.* **112**, 13296–13301 (2015).](http://paperpile.com/b/iTMxNF/0Noxz)

4. [Bayon, G. *et al.*](http://paperpile.com/b/iTMxNF/HaKM) The roles of climate and human land-use in the late Holocene rainforest crisis of Central Africa. [*Earth Planet. Sci. Lett.* **505**, 30–41 (2019).](http://paperpile.com/b/iTMxNF/HaKM)

5. Clist, B. Archaeology in Gabon, 1886–1988. [*African Archaeological Review* **7**, 59–95 (1989).](http://paperpile.com/b/iTMxNF/v1pU)

6. [Ngomanda, A. *et al.*](http://paperpile.com/b/iTMxNF/TmzX) Western equatorial African forest-savanna mosaics: a legacy of late Holocene climatic change? [*Clim. Past* **5**, 647–659 (2009).](http://paperpile.com/b/iTMxNF/TmzX)

7. [Maley, J. *et al.*](http://paperpile.com/b/iTMxNF/1PTv) Late Holocene forest contraction and fragmentation in central Africa. [*Quat. Res.* **89**, 43–59 (2018).](http://paperpile.com/b/iTMxNF/1PTv)

8. [Bayon, G. *et al.*](http://paperpile.com/b/iTMxNF/nBaP) Intensifying weathering and land use in Iron Age Central Africa. [*Science* **335**, 1219–1222 (2012).](http://paperpile.com/b/iTMxNF/nBaP)

9. [Garcin, Y. *et al.* Early anthropogenic impact on Western Central African rainforests 2,600 y ago. *Proc. Natl. Acad. Sci. U. S. A.* **115**](http://paperpile.com/b/iTMxNF/DFzW), 3261–3266 (2018).

10. [Bostoen, K. *et al.*](http://paperpile.com/b/iTMxNF/SPJS) Middle to Late Holocene Paleoclimatic Change and the Early Bantu Expansion in the Rain Forests of Western Central Africa. [*Curr. Anthropol.* **56**, 354–384 (2015).](http://paperpile.com/b/iTMxNF/SPJS)

11. [Neumann, K. *et al.*](http://paperpile.com/b/iTMxNF/Zbgp) Comment on ‘Intensifying weathering and land use in Iron Age Central Africa’. [*Science* vol. 337 1040; author reply 1040 (2012).](http://paperpile.com/b/iTMxNF/Zbgp)

12. [Clist, B. *et al.*](http://paperpile.com/b/iTMxNF/hZ5v) Did human activity really trigger the late Holocene rainforest crisis in Central Africa? [*Proceedings of the National Academy of Sciences of the United States of America* vol. 115 E4733–E4734 (2018).](http://paperpile.com/b/iTMxNF/hZ5v)

13. de Luna, K. M. Conceptualizing vegetation in the Bantu Expansion: Reflections on linguistics in central African history. [*Quat. Int.* **448**, 158–168 (2017).](http://paperpile.com/b/iTMxNF/2ZEGH)

14. Neumann, K. Development of Plant Food Production in the West African Savannas: Archaeobotanical Perspectives. in [*Oxford Research Encyclopedia of African History* (Oxford University Press, 2018).](http://paperpile.com/b/iTMxNF/AlDOl)

15. [Kay, A. U. *et al.*](http://paperpile.com/b/iTMxNF/5qgt2) Diversification, Intensification and Specialization: Changing Land Use in Western Africa from 1800 BC to AD 1500. [*Journal of World Prehistory* (2019) doi:](http://paperpile.com/b/iTMxNF/5qgt2)[10.1007/s10963-019-09131-2](http://dx.doi.org/10.1007/s10963-019-09131-2)[.](http://paperpile.com/b/iTMxNF/5qgt2)

16. Oumar, I., Mariac, C., Pham, J.-L. & Vigouroux, Y. Phylogeny and origin of pearl millet (Pennisetum glaucum [L.] R. Br) as revealed by microsatellite loci. [*Theor. Appl. Genet.* **117**, 489–497 (2008).](http://paperpile.com/b/iTMxNF/3C3b)

17. Manning, K., Pelling, R., Higham, T., Schwenniger, J.-L. & Fuller, D. Q. 4500-Year old domesticated pearl millet (Pennisetum glaucum) from the Tilemsi Valley, Mali: new insights into an alternative cereal domestication pathway. [*J. Archaeol. Sci.* **38**, 312–322 (2011).](http://paperpile.com/b/iTMxNF/e2ZG)

18. [Eggert, M. K. H. *et al.*](http://paperpile.com/b/iTMxNF/zHjc) Pits, graves and grains: Archaeological and archaeobotanical research in southern Cameroon. [*Journal of African Archaeology* **4**, 273–298 (2006).](http://paperpile.com/b/iTMxNF/zHjc)

19. Wotzka., H. P. Ecology and culture of millets in African rainforests: Ancient, historical, and present-day evidence. in [*Trees, Grasses and Crops. People and Plants in Sub-Saharan Africa and Beyond* (eds. Eichhorn, B. & Höhn, A.) 407–429 (Dr. Rudolf Habelt GmbH, 2019).](http://paperpile.com/b/iTMxNF/Y037)

20. Wotzka., H.-P. Experimenteller Anbau von Perlhirse (Pennisetum glaucum) im äquatorialen Regenwald des Inneren Kongobeckens, August–November 2016. in [*Auf dem Holzweg … Eine Würdigung für Ursula Tegtmeier.* (eds. Meurers-Balke, J., Zerl, T. & Gerlach, R.) 269–284 (Propylaeum, 2019).](http://paperpile.com/b/iTMxNF/H7fg)

21. Logan, A. L. & D’Andrea, A. C. Oil palm, arboriculture, and changing subsistence practices during Kintampo times (3600–3200 BP, Ghana). [*Quat. Int.* **249**, 63–71 (2012).](http://paperpile.com/b/iTMxNF/Go6O)

22. Neumann, K., Bostoen, K., Höhn, A., Kahlheber, S. & Tchiengue, B. First farmers in the Central African rainforest: A view from southern Cameroon. [*Quat. Int.* **249**, (2012).](http://paperpile.com/b/iTMxNF/eJK2)

23. Power, R. C., Güldemann, T., Crowther, A. & Boivin, N. Asian Crop Dispersal in Africa and Late Holocene Human Adaptation to Tropical Environments. [*Journal of World Prehistory* **32**, 353–392 (2019).](http://paperpile.com/b/iTMxNF/ALwf)

24. Mercader, J. Foragers of the Congo: The Early Settlement of the Ituri Forest. in [*Under the Canopy: The Archaeology of Tropical Rainforest* (ed. Mercader, J.) 93–116 (Rutgers University Press, 2002).](http://paperpile.com/b/iTMxNF/IIrN)

25. Mercader, J., Marti, R., Wilkins, J. & Fowler, K. The Eastern Periphery of the Yoruba Cultural Sphere. [*Curr. Anthropol.* **47**, 173–184 (2006).](http://paperpile.com/b/iTMxNF/Yp8S)

26. Yasuoka, H. Dense Wild Yam Patches Established by Hunter-Gatherer Camps: Beyond the Wild Yam Question, Toward the Historical Ecology of Rainforests. [*Hum. Ecol. Interdiscip. J.* **41**, 465–475 (2013).](http://paperpile.com/b/iTMxNF/AnbM)

27. [Dounias, E.](http://paperpile.com/b/iTMxNF/mKoT) *The management of wild yam tubers by the Baka pygmies in southern Cameroon*[. (African Study Monographs Supplement 26 135-156, 2001).](http://paperpile.com/b/iTMxNF/mKoT)

28. Bronk Ramsey, C. Bayesian Analysis of Radiocarbon Dates. [*Radiocarbon* **51**, 337–360 (2009).](http://paperpile.com/b/iTMxNF/qiX6)

29. [Reimer, P. J. *et al.*](http://paperpile.com/b/iTMxNF/5mvW) IntCal13 and Marine13 Radiocarbon Age Calibration Curves 0–50,000 Years cal BP. [*Radiocarbon* **55**, 1869–1887 (2013).](http://paperpile.com/b/iTMxNF/5mvW)

30. Eggert, M. K. H. Imbonga and Batalimo: ceramic evidence for early settlement of the equatorial rain forest. [*African Archaeological Review* **5**, 129–145 (1987).](http://paperpile.com/b/iTMxNF/pD9f)

31. Eggert, M. K. H. Central Africa and the archaeology of the equatorial rainforest: reflections on some major topic. in [*The Archaeology of Africa: Food, Metals and Towns* (ed. Shaw, T. , Sinclair, P. , Andah, B. , Okpoko A) (Routledge: London, 1993).](http://paperpile.com/b/iTMxNF/q06m)

32. [Wotzka, H.-P.](http://paperpile.com/b/iTMxNF/of6R) *Studien zur Archäologie des zentralafrikanischen Regenwaldes: die Keramik des inneren Zaïre-Beckens und ihre Stellung im Kontext der Bantu-Expansion*[. (Heinrich-Barth-Institut, 1995).](http://paperpile.com/b/iTMxNF/of6R)

33. Kahlheber, S., Eggert, M. K. H., Seidensticker, D. & Wotzka, H.-P. Pearl Millet and Other Plant Remains from the Early Iron Age Site of Boso-Njafo (Inner Congo Basin, Democratic Republic of the Congo). [*African Archaeological Review* **31**, 479–512 (2014).](http://paperpile.com/b/iTMxNF/gcDU)

34. Eggert, M. K. H. The Archaeology of the Central African Rainforest: Its Current State. in [*The Cambridge World Prehistory* 183–203 (Cambridge University Press, 2014).](http://paperpile.com/b/iTMxNF/Vbyc)

35. [Eggert, R. K.](http://paperpile.com/b/iTMxNF/ieQ4) *Das Wirtschaftssystem der Mongo (Äquatorregion, Zaïre) am Vorabend der Kolonisation. Eine Rekonstruktion*[. (Reimer, 1987).](http://paperpile.com/b/iTMxNF/ieQ4)

36. Nieblas Ramirez, L. Fish bones from Bolondo. Study of the archaeozoological remains of a Nganda in the inner Congo Basin (Tshuapa river DRCongo). (Université Libre de Bruxelles).

37. [Linseele, V.](http://paperpile.com/b/iTMxNF/djjU) *Unpublished Archaeozoology Interim Report for Project Iron Age human subsistence, environment, and climate in the Inner Congo Basin (Democratic Republic of the Congo)*[. (2018).](http://paperpile.com/b/iTMxNF/djjU)

38. Mercader, J., Garralda, M. D., Pearson, O. M. & Bailey, R. C. Eight hundred-year-old human remains from the Ituri tropical forest, Democratic Republic of Congo: the rock shelter site of Matangai Turu Northwest. [*Am. J. Phys. Anthropol.* **115**, 24–37 (2001).](http://paperpile.com/b/iTMxNF/dMPv)

39. Mercader, J., Martı́, R., González, I. J., Sánchez, A. & Garcı́a, P. Archaeological Site Formation in Rain Forests: Insights From the Ituri Rock Shelters, Congo. [*J. Archaeol. Sci.* **30**, 45–65 (2003).](http://paperpile.com/b/iTMxNF/OGWt)

40. Mercader, J. [*et al.* Phytoliths from archaeological sites in the tropical forest of Ituri, Democratic Republic of Congo. *Quat. Res.* **54**, 102–112 (2000).](http://paperpile.com/b/iTMxNF/smgh)

41. Mercader, J. & Brooks, A. S. Across Forests and Savannas: Later Stone Age Assemblages from Ituri and Semliki, Democratic Republic of Congo. [*J. Anthropol. Res.* **57**, 197–217 (2001).](http://paperpile.com/b/iTMxNF/29np)

42. Mercader, J., Garcia-Heras, M. & Gonzalez-Alvarez, I. Ceramic Tradition in the African Forest: Characterisation Analysis of Ancient and Modern Pottery from Ituri, D.R. Congo. [*J. Archaeol. Sci.* **27**, 163–182 (2000).](http://paperpile.com/b/iTMxNF/zKkq)

43. Mercader, J., Rovira, S. & Gómez-Ramos, P. Shared technologies: Forager-farmer interaction and ancient iron metallurgy in the Ituri rainforest, Democratic Republic of Congo. [*Azania: Archaeological Research in Africa* **35**, 107–122 (2000).](http://paperpile.com/b/iTMxNF/Kq4X)

44. Schoeninger, M. J. & Moore, K. Bone stable isotope studies in archaeology. [*J World Prehist* **6**, 247–296 (1992).](http://paperpile.com/b/iTMxNF/7qaA)

45. Katzenberg, M. A. Stable Isotope Analysis: A Tool for Studying Past Diet, Demography, and Life History. in [*Biological Anthropology of the Human Skeleton* (eds. Katzenberg, M. A. & Saunders, S. R.) 411–441 (John Wiley & Sons, Inc., 2008).](http://paperpile.com/b/iTMxNF/Ql6m)

46. Roffet-Salque, M. [*et al.* From the inside out: Upscaling organic residue analyses of archaeological ceramics. *Journal of Archaeological Science: Reports* (2016) doi:](http://paperpile.com/b/iTMxNF/q53Y)[10.1016/j.jasrep.2016.04.005](http://dx.doi.org/10.1016/j.jasrep.2016.04.005)[.](http://paperpile.com/b/iTMxNF/q53Y)

47. O’Leary, M. H. Carbon isotope fractionation in plants. [*Phytochemistry* **20**, 553–567 (1981).](http://paperpile.com/b/iTMxNF/ectp)

48. Ambrose, S. H. Preparation and characterization of bone and tooth collagen for isotopic analysis. [*J. Archaeol. Sci.* **17**, 431–451 (1990).](http://paperpile.com/b/iTMxNF/7Ub5)

49. Calvin, M. & Benson, A. A. The path of carbon in photosynthesis. [*Science* **107**, 476–480 (1948).](http://paperpile.com/b/iTMxNF/FKR2)

50. Hatch, M. D. & Slack, C. R. Photosynthesis by sugar-cane leaves. A new carboxylation reaction and the pathway of sugar formation. [*Biochem. J* **101**, 103–111 (1966).](http://paperpile.com/b/iTMxNF/F0BE)

51. Bonafini, M., Pellegrini, M., Ditchfield, P. & Pollard, A. M. Investigation of the ‘canopy effect’ in the isotope ecology of temperate woodlands. [*J. Archaeol. Sci.* **40**, 3926–3935 (2013).](http://paperpile.com/b/iTMxNF/uEZu)

52. van der Merwe, N. J. & Medina, E. The canopy effect, carbon isotope ratios and foodwebs in amazonia. [*J. Archaeol. Sci.* **18**, 249–259 (1991).](http://paperpile.com/b/iTMxNF/nlnb)

53. Ambrose, S. H. & Norr, L. Experimental Evidence for the Relationship of the Carbon Isotope Ratios of Whole Diet and Dietary Protein to Those of Bone Collagen and Carbonate. in [*Prehistoric Human Bone: Archaeology at the Molecular Level* (eds. Lambert, J. B. & Grupe, G.) 1–37 (Springer Berlin Heidelberg, 1993).](http://paperpile.com/b/iTMxNF/SG3w)

54. O’Connell, T. C., Kneale, C. J., Tasevska, N. & Kuhnle, G. G. C. The Diet-Body Offset in Human Nitrogen Isotopic Values: A Controlled Dietary Study. [*Am. J. Phys. Anthropol.* **149**, 426–434 (2012).](http://paperpile.com/b/iTMxNF/N5SYt)

55. Schoeninger, M. J., DeNiro, M. J. & Tauber, H. Stable nitrogen isotope ratios of bone collagen reflect marine and terrestrial components of prehistoric human diet. [*Science* **220**, 1381–1383 (1983).](http://paperpile.com/b/iTMxNF/DWycq)

56. Schoeninger, M. J. & DeNiro, M. J. Nitrogen and carbon isotopic composition of bone collagen from marine and terrestrial animals. [*Geochim. Cosmochim. Acta* **48**, 625–639 (1984).](http://paperpile.com/b/iTMxNF/dvgYg)

57. Fahy, E., Deter, C., Pitﬁeld, R., Miszkiewicz, J. J. & Mahoney, P. Bone deep: Variation in stable isotope ratios and histomorphometric measurements of bone remodelling within adult humans. [*J. Archaeol. Sci.* **87**, 10–16 (2017).](http://paperpile.com/b/iTMxNF/E1R8)

58. Hedges, R. E. M., Clement, J. G., Thomas, C. D. L. & O’connell, T. C. Collagen turnover in the adult femoral mid-shaft: modeled from anthropogenic radiocarbon tracer measurements. [*Am. J. Phys. Anthropol.* **133**, 808–816 (2007).](http://paperpile.com/b/iTMxNF/tkWJ)

59. Eerkens, J. W., Berget, A. G. & Bartelink, E. J. Estimating weaning and early childhood diet from serial micro-samples of dentin collagen. [*J. Archaeol. Sci.* **38**, 3101–3111 (2011).](http://paperpile.com/b/iTMxNF/Z5oE5)

60. [Hillson, S.](http://paperpile.com/b/iTMxNF/Cgap) *Dental Anthropology*[. (Cambridge University Press, 1996).](http://paperpile.com/b/iTMxNF/Cgap)

61. Buchmann, N. & Ehleringer, J. R. CO2 concentration profiles, and carbon and oxygen isotopes in C3 and C4 crop canopies. [*Agric. For. Meteorol.* **89**, 45–58 (1998).](http://paperpile.com/b/iTMxNF/i96Y)

62. Dansgaard, W. Stable isotopes in precipitation. [*Tell’Us* **16**, 436–468 (1964).](http://paperpile.com/b/iTMxNF/FphT)

63. Levin, N. E., Cerling, T. E., Passey, B. H., Harris, J. M. & Ehleringer, J. R. A stable isotope aridity index for terrestrial environments. [*Proc. Natl. Acad. Sci. U. S. A.* **103**, 11201–11205 (2006).](http://paperpile.com/b/iTMxNF/zSHR)

64. Krigbaum, J. Neolithic subsistence patterns in northern Borneo reconstructed with stable carbon isotopes of enamel. [*Journal of Anthropological Archaeology* **22**, 292–304 (2003).](http://paperpile.com/b/iTMxNF/jLEu)

65. [Roberts, P.](http://paperpile.com/b/iTMxNF/f3xy) *et al.* [Direct evidence for human reliance on rainforest resources in late Pleistocene Sri Lanka. *Science* **347**, 1246–1249 (2015).](http://paperpile.com/b/iTMxNF/f3xy)

66. [Roberts, P.](http://paperpile.com/b/iTMxNF/oP5Q) *et al.* [Fruits of the forest: Human stable isotope ecology and rainforest adaptations in Late Pleistocene and Holocene (∼36 to 3 ka) Sri Lanka. *J. Hum. Evol.* **106**, 102–118 (2017).](http://paperpile.com/b/iTMxNF/oP5Q)

67. Reber, E. A. & Evershed, R. P. How Did Mississippians Prepare Maize? The Application of Compound-Specific Carbon Isotope Analysis to Absorbed Pottery Residues From Several Mississippi Valley Sites. [*Archaeometry* **46**, 19–33 (2004).](http://paperpile.com/b/iTMxNF/aeVU)

68. Reber, E. A. & Evershed, R. P. Identification of maize in absorbed organic residues: a cautionary tale. [*J. Archaeol. Sci.* **31**, 399–410 (2004).](http://paperpile.com/b/iTMxNF/HaMv)

69. [Heron, C.](http://paperpile.com/b/iTMxNF/T0aH) *et al.* [First molecular and isotopic evidence of millet processing in prehistoric pottery vessels. *Sci. Rep.* **6**, 38767 (2016).](http://paperpile.com/b/iTMxNF/T0aH)

70. [Heiss, A. G.](http://paperpile.com/b/iTMxNF/ajgf) *et al.* [State of the (t)art. Analytical approaches in the investigation of components and production traits of archaeological bread-like objects, applied to two finds from the Neolithic lakeshore settlement Parkhaus Opéra (Zürich, Switzerland). *PLoS One* **12**, e0182401 (2017).](http://paperpile.com/b/iTMxNF/ajgf)

71. Fuller, D. Q. & Gonzalez Carretero, L. The archaeology of Neolithic cooking traditions: archaeobotanical approaches to baking, boiling and fermenting. [*AI* **21**, 109 (2018).](http://paperpile.com/b/iTMxNF/cLtF)

72. Eggert, M. K. H. Imbonga und Lingonda: Zur frühesten Besiedlung des zentralafrikanischen Regenwaldes. [*Beiträge zur Allgemeinen und Vergleichenden Archäologie* **6**, 247–288 (1984).](http://paperpile.com/b/iTMxNF/u8vJ)
