# Supplementary material for: Isotopic and microbotanical insights into Iron Age agricultural reliance in the Central African rainforest
Source: Commun Biol. 2020 Oct 27;3:619. doi: 10.1038/s42003-020-01324-2 (PMC7591565; doi:10.1038/s42003-020-01324-2)
Supplement: Supplementary file 3 — Description of Additional Supplementary Files [file 42003_2020_1324_MOESM3_ESM.pdf]

Description of Additional Supplementary Files

File Name: Supplementary Data 1
Description:
Sheet 1: Sample and standard EA-IRMs data (session 1). Samples with Amplitude m/z 44
Sheet 2: Sample and standard EA-IRMs data (session 2). Samples with Amplitude m/z 44
Sheet 3: Calibration of the results from Session 1 and Session 2.
Sheet 4: Uncertainty calculations for $\delta^{13}C$.
Sheet 5: Uncertainty calculations for $\delta^{15}N$.
Sheet 6: Uncertainty calculations for %C. Sheet 7: Uncertainty calculations for %N.
Sheet 7: Uncertainty calculations for %N
