# Supplementary material for: Isotopic and microbotanical insights into Iron Age agricultural reliance in the Central African rainforest
Source: Commun Biol. 2020 Oct 27;3:619. doi: 10.1038/s42003-020-01324-2 (PMC7591565; doi:10.1038/s42003-020-01324-2)
Supplement: Supplementary file 5 — Reporting Summary [file 42003_2020_1324_MOESM5_ESM.pdf]

nature research

Corresponding author(s): Madeleine Bleasdale, Patrick Roberts

Last updated by author(s): 2020/08/25

Reporting Summary

Nature Research wishes to improve the reproducibility of the work that we publish. This form provides structure for consistency and transparency in reporting. For further information on Nature Research policies, see Authors & Referees and the Editorial Policy Checklist .

Statistics

For all statistical analyses, confirm that the following items are present in the figure legend, table legend, main text, or Methods section.

n/a	Confirmed	
☐	☒	The exact sample size (*n*) for each experimental group/condition, given as a discrete number and unit of measurement
☐	☒	A statement on whether measurements were taken from distinct samples or whether the same sample was measured repeatedly
☒	☐	The statistical test(s) used AND whether they are one- or two-sided<br>*Only common tests should be described solely by name; describe more complex techniques in the Methods section.*
☐	☒	A description of all covariates tested
☒	☐	A description of any assumptions or corrections, such as tests of normality and adjustment for multiple comparisons
☐	☒	A full description of the statistical parameters including central tendency (e.g. means) or other basic estimates (e.g. regression coefficient) AND variation (e.g. standard deviation) or associated estimates of uncertainty (e.g. confidence intervals)
☐	☒	For null hypothesis testing, the test statistic (e.g. *F*, *t*, *r*) with confidence intervals, effect sizes, degrees of freedom and *P* value noted<br>*Give P values as exact values whenever suitable.*
☒	☐	For Bayesian analysis, information on the choice of priors and Markov chain Monte Carlo settings
☒	☐	For hierarchical and complex designs, identification of the appropriate level for tests and full reporting of outcomes
☐	☒	Estimates of effect sizes (e.g. Cohen's *d*, Pearson's *r*), indicating how they were calculated

Our web collection on statistics for biologists contains articles on many of the points above.

Software and code

Policy information about availability of computer code

Data collection	n/a
Data analysis	R Core Team, 2019 - Free Program R Software

For manuscripts utilizing custom algorithms or software that are central to the research but not yet described in published literature, software must be made available to editors/reviewers. We strongly encourage code deposition in a community repository (e.g. GitHub). See the Nature Research guidelines for submitting code & software for further information.

Data

Policy information about availability of data

All manuscripts must include a data availability statement. This statement should provide the following information, where applicable:

- Accession codes, unique identifiers, or web links for publicly available datasets
- A list of figures that have associated raw data
- A description of any restrictions on data availability

All raw data is available in the main text and supplementary information

Field-specific reporting

Please select the one below that is the best fit for your research. If you are not sure, read the appropriate sections before making your selection.

☐ Life sciences ☐ Behavioural & social sciences ☒ Ecological, evolutionary & environmental sciences

For a reference copy of the document with all sections, see nature.com/documents/nr-reporting-summary-flat.pdf

Ecological, evolutionary & environmental sciences study design

All studies must disclose on these points even when the disclosure is negative.

Study description

Stable carbon, oxygen and nitrogen isotope analysis was applied to archaeological human and animal tooth enamel and bone collagen from four archaeological sites in the Democratic Republic of the Congo. This was done to investigate dietary consumption in the Congo Basin during the Iron Age and assess human reliance on incoming domestic cereals.

Research sample

Bone collagen samples were selected from humans previously excavated from the archaeological sites of Longa, Imbonga, Bolondo and Matangai Turu Northwest in the Congo Basin. δ13C and δ15N measurements of bone collagen were made to investigate dietary consumption, largely protein sources such as meat, from the sites of study. To provide a baseline, collagen was also analysed from a range of wild and domestic fauna from the site of Bolondo, including crocodile, antelope, goat and dog. In addition tooth enamel was samples for δ18O and δ13C from humans and fauna to explore the consumption of wild plant sources (C3) compared to incoming domestic crops (C4) such as pearl millet. Stable carbon and nitrogen isotope analyses were also performed on a number of charred food remains from Bolondo to further investigate the processing of different foodstuffs. For a single individual from Matangai Turu Northwest, dental calulus (calcified dental plaque) was removed and analyzed using microscopy to identify plant phytoliths and starch granules entrapped in the calculus.

Sampling strategy

Samples were selected for stable carbon and oxygen isotope analysis of tooth enamel and stable carbon and nitrogen isotope analysis of bone collagen from the available fossil material at the archaeological sites of Longa, Imbonga, Bolondo and Matangai Turu Northwest in the Congo Basin. All teeth or tooth fragments were cleaned using air-abrasion to remove any external material. Enamel powder for bulk analysis was obtained using gentle abrasion with a diamond-tipped drill along the full length of the buccal surface in order to ensure a representative measurement for the entire period of enamel formation. All bone fragments were cleaned with air-abrasion to remove any soil.

Data collection

Bone samples were cleaned by abrasion using a sandblaster. Samples were demineralised in 0.5M HCl for 1-7 days and rinsed three times with H2O. The residue was gelatinised in pH3 HCl at 70°C for 48 hours and the solution Ezee-filtered. Samples were lyophilised in a freeze dryer for 48hrs. 1.0 mg of purified collagen was weighed, in duplicate, into tin capsules for analysis. The δ13C and δ15N ratios of the bone collagen were determined using a Thermo Scientific Flash 2000 Elemental Analyser coupled to a Thermo Delta V Advantage mass spectrometer at the Isotope Laboratory, MPI-SHH, Jena. Isotopic values are reported as the ratio of the heavier isotope to the lighter isotope (13C/12C or 15N/14N) as δ values in parts per mill (‰) relative to international standards, VPDB for δ13C and atmospheric N2 (AIR) for δ15N. Results were calibrated against international standards of (IAEA-CH-6: δ13C = -10.80 ± 0.47 ‰, IAEA-N-2: δ15N = 20.3 ± 0.2 ‰, and USGS40: δ13C = -26.38 ± 0.042 ‰, δ15N = 4.5 ± 0.1 ‰) and a laboratory standard (fish gelatin: δ13C = ~ -15.1 ‰, δ15N = ~14.3 ‰). Based on replicate analyses long-term machine error over a year is ± 0.2‰ for δ13C and ±0.2‰ for δ15N. Overall measurement precision was studied through the measurement of repeats of fish gelatin (n= 80, ± 0.2‰ for δ13C and±0.2‰ for δ15N). Analysis was performed by Madeleine Bleasdale, Jana Zech, Sara Marzo, Bianca Fiedler and Patrick Roberts.

Enamel was pretreated to remove organic or secondary carbonate contaminants. Samples were washed in 1.5% sodium hypochlorite for 60 minutes, followed by three rinses in purified H2O and centrifuging, before 0.1M acetic acid was added for 10 minutes, followed by another three rinses in purified H2O. Following reaction with 100% phosphoric acid, gases evolved from the samples were analyzed to stable carbon and oxygen isotopic composition using a Thermo Gas Bench 2 connected to a Thermo Delta V Advantage Mass Spectrometer at the Department of Archaeology, Max Planck Institute for the Science of Human History. Carbon (δ13C) and oxygen (δ18O) stable isotope values were calibrated against international standards IAEA NBS 18 (δ13C -5.014 ± 0.032 ‰, δ18O -23.2±0.1 ‰), IAEA 603 (δ13C +2.46±0.01 ‰, δ18O -2.37±0.04 ‰), IAEA CO8 (δ13C -5.764±0.032 ‰, δ18O -22.7±0.2 ‰), and USGS44 (δ13C = ~ -42.1 ‰) registered by the International Atomic Energy Agency. Machine error based on the analyses of standards is ± 0.1‰ for δ13C and±0.2‰ for δ18O. Overall measurement precision was assessed through repeat measurements of MERCK CaCO3 (n= 20, ± 0.2‰ for δ13C and±0.2‰ for δ18O, δ13C = ~ -40.6 ‰, δ18O= ~ -13.3 ‰) and an in-house equid tooth standard (n=10, ± 0.3‰ for δ13C and±0.2‰ for δ18O). Analysis was performed by Madeleine Bleasdale, Jana Zech, Sara Marzo, Bianca Fiedler and Patrick Roberts.

Charred fragments classified as food remains were retrieved from flotation samples from Bolondo. 2-3 mg of each sample was weighed into tin capsules for isotope analysis. δ13C and δ15N ratios of the charred food fragments were determined using a Thermo MAT 253 continuous flow isotope ratio mass spectrometer coupled to a Thermo Flash 1,112 Series elemental analyser in the Institut für Geowissenschaften, Goethe-Universität, Frankfurt am Main, Germany. Isotopic data are provided in Supplementary Table 6. The carbon contents of the samples were calculated based on the area under the CO2 peak relative to the weight of the sample, calibrated using IAEA-CH-7. Stable carbon isotope values were calibrated to the VPDB scale using IAEA-C-7 (δ13C –32.15 ± 0.05 ‰) and IAEA-USGS24 (δ13C –16.05 ± 0.04 ‰). Measurement uncertainty in δ13C values was monitored using three in-house standards: LEU (DL-leucine, δ13C –28.3 ± 0.1 ‰), GLU (DL-glutamic acid monohydrate, δ13C –10.4 ± 0.1 ‰) and MIL (millet flour from a single panicle from a plot in Senegal, δ13C –10.2 ± 0.1 ‰) (Supplementary Tables 7–9). Precision (u(Rw)) was determined to be ± 0.06 ‰, accuracy or systematic error (u(bias)) was ± 0.11 ‰ and the total analytical uncertainty in δ13C values was estimated to be ± 0.13 ‰ using the equation presented in Supplementary material (Supplementary Table 10–13).The nitrogen contents of the samples were calculated based on the area under the N2 peak relative to the weight of the sample, calibrated using IAEA-N2. Stable nitrogen isotope values were calibrated to the AIR scale using IAEA-N-1 (δ15N 0.4 ± 0.2 ‰) and IAEA-N-2 (δ15N 20.3 ± 0.2 ‰). Measurement uncertainty in δ15N values was monitored using three in-house standards: LEU (DL-leucine, δ15N 6.5 ± 0.4 ‰), GLU (DL-glutamic acid monohydrate, δ15N –1.9 ± 0.1 ‰) and MIL (millet flour from a single panicle from a plot in Senegal, δ15N 3.1 ± 0.6 ‰). u(Rw) was determined to be ± 0.18 ‰, u(bias) was ± 0.59 ‰ and the total analytical uncertainty in δ15N values was estimated to be ± 0.61 ‰. Analysis was performed by Amy Styring.

Dental calculus was removed from three mandibular molars: M1-M3. Images of the mineralised plaque prior to removal, as well as those from contaminant starch granules and phytoliths are published elsewhere.Microbotanical materials released from the calcified

matrix after decalcification following previously published decontamination protocols were identified. In addition, microbotanicals still trapped in the calculus matrix were identified following previously published morphometric classification criteria for the identification of ancient starch from sub-Saharan plants. Analysis was performed by Julio Mercader.

Timing and spatial scale	The analyses performed were not time dependent.
Data exclusions	No data were excluded from the analyses.
Reproducibility	Based on replicate analyses long-term machine error over a year is ± 0.2‰ for δ13C and±0.2‰ for δ15N. Overall measurement precision was studied through the measurement of repeats of fish gelatin (n= 80, ± 0.2‰ for δ13C and±0.2‰ for δ15N). Machine error based on the analyses of standards is ± 0.1‰ for δ13C and±0.2‰ for δ18O. Overall measurement precision was assessed through repeat measurements of MERCK CaCO3 (n= 20, ± 0.2‰ for δ13C and±0.2‰ for δ18O, δ13C = ~ -40.6 ‰, δ18O= ~ -13.3 ‰) and an in-house equid tooth standard (n=10, ± 0.3‰ for δ13C and±0.2‰ for δ18O).
Randomization	A mixture of wild and domestic fauna from Bolondo were selected for analyses based on perservation of skeletal elements. Bone and teeth were sampled from human burials from Bolondo, Imbonga, Longa and Matangai Turu Northwest.
Blinding	Blinding is not relevant as the experiments performed are based on all available material.

Did the study involve field work? ☐ Yes ☒ No

Reporting for specific materials, systems and methods

We require information from authors about some types of materials, experimental systems and methods used in many studies. Here, indicate whether each material, system or method listed is relevant to your study. If you are not sure if a list item applies to your research, read the appropriate section before selecting a response.

Materials & experimental systems

n/a	Involved in the study	
☒	☐	Antibodies
☒	☐	Eukaryotic cell lines
☐	☒	Palaeontology
☒	☐	Animals and other organisms
☒	☐	Human research participants
☒	☐	Clinical data

Methods

n/a	Involved in the study	
☒	☐	ChIP-seq
☒	☐	Flow cytometry
☒	☐	MRI-based neuroimaging

Palaeontology

Specimen provenance	Archaeological samples came from the sites of Longa, Imbonga, Bolondo and Matangai Turu Northwest in the Congo Basin. Samples were collected during previous excavations led by Manfred Eggert, Hans-Peter Wotzka and Julio Mercader.
Specimen deposition	Left over material from the analyses are deposited at the Isotope Laboratory of the Max Planck Institute for the Science of Human History, Jena, Germany with the sample codes BLD IMB LON MTNW. Samples will be returned to the University of Cologne and eventually the DRC.
Dating methods	Archaeological human bone samples (n=6) were sent to the Scottish Universities Environmental Research Centre AMS Laboratory, Glasgow (SUERC, Lab ID: GU). Radiocarbon ages were calibrated to calendar timescale using OxCal 4 and IntCal13 atmospheric calibration curve.

☒ Tick this box to confirm that the raw and calibrated dates are available in the paper or in Supplementary Information.
